# Variability in micro RNA (miRNA) abundance, speciation and complexity amongst different human populations and potential relevance to Alzheimer's disease (AD)

**DOI:** 10.3389/fncel.2013.00133

**Published:** 2013-08-27

**Authors:** Walter J. Lukiw

**Affiliations:** Department of Neurology, Neuroscience and Ophthalmology, LSU Neuroscience Center, Louisiana State University Health Sciences CenterNew Orleans, LA, USA

**Keywords:** miRNA profiling, miRNA speciation, Alzheimer's disease, human populations, caucasian american, african american, superior temporal lobe neocortex, human biochemical individuality

Toward the end of his 1976 book entitled “*Vitamin C and the Common Cold*” Linus Pauling included an interesting chapter on “*human biochemical individuality*” that defined some important parameters on individual human genotypic versus phenotypic variation, based in part on studies from hemoglobin genetics (Pauling, 1976). That chapter provided theoretical calculations and novel insight on genomic diversity, discussing, that when any genetic characteristic is analyzed in a sampling of 100 human beings, wide ranges in values are invariably observed. The *“normalcy”* range was defined as that in which 95% of those “normal values” lie, while the remaining 5% are described as “abnormal.” Defining *“normalcy”* on a larger scale becomes a bit trickier however, and Pauling proposed that if we assume that 500 characters are independently inherited, then we can calculate that there is only a small chance, 10%, that one person in the entire global population would be normal (with respect to each of these 500 characters). If we assume that there are about 26,600 human genes available to be expressed in each cell and that each gene is responsible for *at least one* inherited trait or genetic function (a number that is probably vastly underestimated), in a global human population exceeding 7 billion it then becomes exceedingly difficult to define “*human genetic normalcy*.” These ideas form the basis for the evolving concept of *“human genetic individuality”* and our ongoing efforts to better understand the genotypic basis of human phenotypic diversity in both health and disease (Li et al., 2010; Raj et al., 2010; Lukiw, 2012a,b; Olson, 2012; this paper). More recently, large population studies have analyzed the contribution of variability in gene expression, including the impact of genetic mutations, to “*human genetic normalcy*,” *“human genetic individuality*,” phenotype, susceptibility to disease and related parameters that include the general redundancy in human gene expression that may directly impact the genetic evolution of the human species (Colangelo et al., 2002; Li et al., 2010; Zheng et al., 2011; Lukiw, 2012a,b; Ginsberg et al., 2012; Raj et al., 2012; Hulse and Cai, 2013). This “Opinion paper” addresses an observed variability in micro RNA (miRNA) abundance, speciation and complexity in Alzheimer's disease (AD), a common, progressive neurological disorder unique to the aging human brain whose incidence is approaching epidemic proportions (Alzheimer Association, 2013). Here we define miRNA abundance as how much of each individual miRNA species is present, miRNA speciation as what individual miRNA species are present, and miRNA complexity as the pattern of miRNA abundance and speciation representative of a particular physiological or pathophysiological state.

One overwhelming observation that becomes apparent in gene expression analysis is the vast variability in gene expression patterns in cells and tissues derived from different human populations - these being most noticeable from Northern-, RT-PCR- and high density array-based measurements on both messenger RNA (mRNA) and miRNA abundance, speciation and complexity in defined brain anatomical regions from different human samples. These studies have been very valuable since the profiling of mRNA and/or miRNA can provide a powerful “snapshot” into the physiological status of a human cell or tissue in health and disease, and may even be predictive for the prognosis and/or diagnosis for the future outcomes of other AD patients. Steady-state mRNA and miRNA levels from different individuals clearly indicates that the abundance and speciation of these RNAs within clearly defined anatomical regions can significantly differ between samples analyzed, suggesting that genetic variation and extraneous effects, including age, gender, body mass index (BMI), apolipoprotein E (ApoE), beta-amyloid cleavage enzyme (BACE) and other AD-relevant allele status, life-style and intrinsic population effects can influence the profile of mRNA or miRNA abundance and complexity (Colangelo et al., 2002; Cui et al., 2005; Lukiw, 2007; Lukiw and Pogue, 2007; Williams et al., 2007; Sethi and Lukiw, 2009; Ginsberg et al., 2012). These patterns are further complicated by tissue acquisition and quality control parameters that include agonal effects, the analytical approach, and the death-to-brain freezing interval for post-mortem human tissues (McLachlan et al., 1989; Cui et al., 2005; Williams et al., 2007; Sethi and Lukiw, 2009). Agonal effects include the circumstances accompanying brain death, such as whether or not fever (i.e., heat shock) was present, whether there was interceding or accompanying illness including, commonly, pneumonia or cerebrovascular disease, and other pathophsyiological or interrelated procedural or clinical factors (Sethi and Lukiw, 2009; Raj et al., 2010; Hulse and Cai, 2013).

To illustrate one important example is the miRNA abundance and speciation of a small family of inducible, NF-kB-sensitive miRNAs in two different American populations—Caucasian Americans and African Americans afflicted with AD (Figure 1). A pathogenic quintet of up-regulated miRNAs have been described and partially characterized, and these include miRNA-9, miRNA-34a, miRNA-125b, miRNA-146a and miRNA-155, which have been shown to be involved in chronic inflammatory degeneration by many independent groups in multiple human diseases with a progressive inflammatory and degenerative component (Lukiw, 2007; Williams et al., 2007; Wang et al., 2009; Culpan et al., 2011; Lukiw et al., 2011; Hu et al., 2012; Iyer et al., 2012; Saba et al., 2012; Lukiw, 2013; Nussbaum, 2013; Zhao et al., 2013). In AD these five up-regulated miRNAs appear to play important roles in the down-regulation of brain gene expression normally involved in the brain's neurotrophic support, synaptogenesis, the innate-immune response, NF-kB-mediated inflammatory signaling and amyloidogenesis (Cui et al., 2005; Sethi and Lukiw, 2009; Lukiw, 2012a,b; Zhao et al., 2013). Preliminary data indicates that greater general abundance in the expression of these five miRNAs may in part explain differences in the incidence, course and/or severity of AD amongst elderly Caucasian American, African American, Hispanics and other minority populations. Interestingly, when comparing AD in human populations, African Americans and Hispanics appear to have an increased frequency and severity of AD when compared to Caucasians, which may be independent of their *APOE* genotype (Tang et al., 1998; Shadlen et al., 1999; Reitz et al., 2013). The current results further suggest that in contrast to a recent mRNA-based study of genetic homogeneity in aging humans (Colantuoni et al., 2011), increased abundance of pathological miRNAs in progressive neurodegenerative disorders may reflect gene expression patterns highly characteristic of the AD process in certain human populations. These results further underscore basic differences in miRNA versus mRNA function, in accordance with their differential modes of generation, processing and signaling in development, aging and disease. As both mRNA and miRNA are intrinsically unstable molecules with short half-lives, differential studies using only high quality, high RIN value, short post-mortem interval (PMI) mRNA and miRNA may be very useful in furthering our understanding of AD epidemiology, and ultimately also be of use diagnostically and therapeutically in the clinical management of this common neurological disorder (Espino and Lewis, 1998; Froehlich et al., 2001; Cowley et al., 2009; Sethi and Lukiw, 2009; Venketasubramanian et al., 2010; Lukiw, 2013; Reitz et al., 2013).

**Figure 1:**
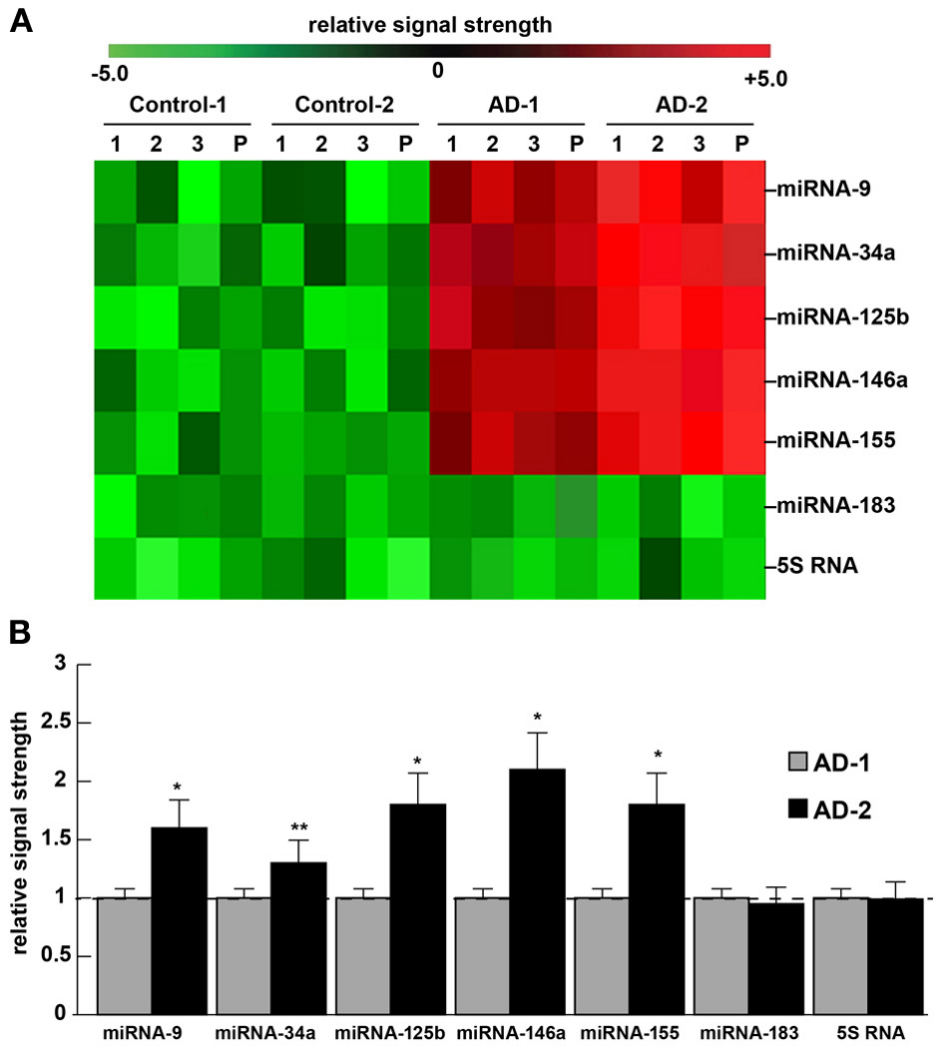
**Incidence of miRNA abundance, speciation and complexity for 5 NF-kB-sensitive pro-inflammatory miRNAs in the superior temporal lobe (Brodmann Area A22) of 2 different human populations: The current explosion in miRNA profiling of human disease, including neuro-degenerative diseases such as Alzheimer's disease (AD), underscores “human genetic individuality.”** Preliminary data suggests that there is considerable variation in miRNA abundance, speciation and complexity in human populations, and variation in miRNA abundance amongst individuals or populations may be a reflection of their individual genetic-based susceptibility to disease incidence or severity. **(A)** depicts a representative color-coded cluster diagram for 2 control and 2 selected “AD” populations; control-1 miRNA signals are derived from Caucasian Americans [age mean ± 1 standard deviation (*SD*) = 75.5 ± 8.4 year] and control-2 miRNA signals (age mean ± 1 *SD* = 76.1 ± 7.8 year) are derived from African Americans; similarly AD-1 miRNA signals are derived from Caucasian Americans [age mean ± 1 SD = 77.4 ± 7.5 year] with AD, and AD-2 miRNA signals are derived from African Americans (age mean ± 1 *SD* = 76.6 ± 8.2 year) with AD; all AD cases were for moderate-to-advanced stages of AD. Because, as single stranded ribonucleotides, miRNAs appear to have a relatively short half-life, all PMIs had a mean of 2.1 h or less (Sethi and Lukiw, 2009; Cui et al., 2010); there were no significant differences in age, PMI, ApoE allele status, RNA quality (all RIN values were 8.1–9.0) or yield between the control or AD groups (*p* > 0.05, ANOVA), or between the Caucasian and African American groups; note the higher general expression for miRNA-9, miRNA-34a, miRNA-125b, miRNA-146a and miRNA-155 (a) for all AD cases over controls and (b) for AD-2 versus AD-1; miRNAs in all AD cases were compared to 2 unchanging internal controls miRNA-183 and 5S RNA in the same brain sample; the numbers “1,” “2” and “3” are from individual control or AD cases; the letter “P” (also analyzed in **B**); using miRNA arrays, in Caucasian Americans miRNA-9, miRNA-34a, miRNA-125b, miRNA-146a and miRNA-155 were found to be up-regulated an average of 1.5-to-3.5 fold over age-matched controls, in African Americans this same group of miRNAs averaged an up-regulation of 3-to-5-fold over age-matched controls. **(B)** (bar graph) depicts quantitative results using RT-PCR, comparing AD-1 miRNA abundance [AD, (*N* = 8) relative to control miRNA (*N* = 8) signals; Caucasian Americans, set to 1.0 (for ease of comparison; dashed horizontal line)] to AD-2 miRNA abundance [AD (*N* = 8) relative to control miRNA (*N* = 8) signals; African Americans]; the data is suggestive of significantly higher miRNA abundance for these 5 potentially pathogenic miRNAs in the AD-2 group which may, in part, form a molecular-genetic basis for the predisposition of African Americans, and perhaps other ethnic groups, to different incidences of AD-type neuropathology, including variations in dementia development, severity, age of onset, progression, course and epidemiology (Espino and Lewis, 1998; Tang et al., 1998; Shadlen et al., 1999; Cui et al., 2010; Venketasubramanian et al., 2010; Reitz et al., 2013; this paper); ^*^p < 0.01; ^**^p < 0.05 (ANOVA). As further discussed in the text, selective differences in miRNA abundance may be useful in AD diagnosis and individualistic therapeutic strategies, to tailor more effective clinical treatment for AD and other progressive, age-related neurological disorders of the human CNS.

Variation in miRNA patterns lends further strength to the idea that AD is not a single, definable neurological disease entity such as sickle-cell anemia (Pauling, 1976), but rather a syndrome. Syndromes are typically a collection of biomedical symptoms known to frequently appear together, but without a known or well-defined cause. For example, the single point mutation (GAG->GTG) in the beta-hemoglobin chain that changes a glutamate-to-valine, thus generating mutant hemoglobin causes sickle cell anemia virtually 100% of the time; there is no similar single nucleotide change in any gene product known that associates with, or causes AD. Rather, in AD it appears that multiple interdependent neurogenetic, neurochemical and neurobiological insults progressively accumulate and chronically drive oxidative stress, apoptosis, neuronal cell death, synaptic loss and the age-related accumulation of senile plaque and neurofibrillary tangles, the pathological hall marks of AD. Obviously neurons can die at different rates from diverse pathogenic mechanisms, and different types of neurons have varied susceptibilities and thresholds to neurotoxic insults. The recently appreciated contribution of the microbiome to human systemic physiology may very well also be involved in homeostasis, health and diseases including AD (Kostic et al., 2013). Further, epigenetic and environmental factors such as diet, exercise, stress and life-style, factors which are known to impact both AD pathology and gene expression patterns, are highly variable amongst different human populations. Large rigorous population-based studies involving these multiple risk parameters still need to be compiled, researched and analyzed (Williams et al., 2007; Nussbaum, 2013).

Lastly, much independently derived data comparable to that shown in Figure 1 supports the idea that the genetics and epigenetics of AD varies widely amongst different human populations with different genetic backgrounds, and these observations are in accordance with the concept of *“human genetic individuality*.” If molecular-genetic and epigenetic profiles of AD brain samples are any indication of AD phenotypic variation then there may be real and significant inter-ethnic differences in AD epidemiology, incidence, disease course and progression. This further suggests that an equally wide variety of diagnostic and individualistic prevention and treatment strategies will be required to more effectively address such progressive, age-related neurological disorders of the human CNS, including the implementation of novel combinatorial therapeutic strategies such as anti-NF-kB and anti-miRNA approaches that have not yet been considered (Lukiw, 2013).

## References

[B1] Alzheimer Association. (2013). Alzheimer's Disease Facts and Figures Statistical Resource. Available online at: http://www.alz.org/downloads/Facts_Figures_2013.pdf

[B2] Colangelo V. Schurr J. Ball M. J. Pelaez R. P. Lukiw W. J. (2002). Gene expression profiling of 12633 genes in Alzheimer hippocampal CA1: transcription and neurotrophic factor down-regulation and up-regulation of apoptotic and pro-inflammatory signaling. J. Neurosci. Res. 70, 462–473 10.1002/jnr.10351 12391607

[B3] Colantuoni C. Lipska B. K. Ye T. Hyde T. M. Tao R. Leek J. T. (2011). Temporal dynamics and genetic control of transcription in the human prefrontal cortex. Nature 478, 19–23 10.1038/nature10524 22031444 PMC3510670

[B4] Cowley M. J. Cotsapas C. J. Williams R. B. Chan E. K. Pulvers J. N. Liu M. Y. (2009). Intra- and inter-individual genetic differences in gene expression. Mamm. Genome 20, 281–295 10.1007/s00335-009-9181-x 19424753 PMC2690833

[B5] Cui J. G. Zhao Y. Lukiw W. J. (2005). Isolation of high spectral quality messenger RNA (mRNA) using run-on gene transcription; application to gene expression profiling of human brain. Cell. Mol. Neurobiol. 25, 789–794 10.1007/s10571-005-4035-x 16075392 PMC11529491

[B6] Cui J. G. Li Y. Y. Zhao Y. Bhattacharjee S. Lukiw W. J. (2010). Differential regulation of interleukin-1 receptor-associated kinase-1 (IRAK-1) and IRAK-2 by microRNA-146a and NF-kappaB in stressed human astroglial cells and in Alzheimer disease. J. Biol. Chem. 285, 38951–38960 10.1074/jbc.M110.178848 20937840 PMC2998119

[B7] Culpan D. Kehoe P. G. Love S. (2011). Tumour necrosis factor-α (TNF-α) and miRNA expression in frontal and temporal neocortex in Alzheimer's disease and the effect of TNF-α on miRNA expression *in vitro*. Int. J. Mol. Epidemiol. Genet. 2, 156–162 21686130 PMC3110390

[B8] Espino D. V. Lewis R. (1998). Dementia in older minority populations; issues of prevalence, diagnosis, and treatment. Am. J. Geriatr. Psychiatry 6, S19–S25 10.1097/00019442-199821001-00003 9581217

[B9] Froehlich T. E. Bogardus S. T. Jr. Inouye S. K. (2001). Dementia and race: are there differences between African Americans and Caucasians. J. Am. Geriatr. Soc. 49, 477–484 10.1046/j.1532-5415.2001.49096.x 11347796

[B10] Ginsberg S. D. Alldred M. J. Che S. (2012). Gene expression levels assessed by CA1 pyramidal neuron and regional hippocampal dissections in Alzheimer's disease. Neurobiol. Dis. 45, 99–107 10.1016/j.nbd.2011.07.013 21821124 PMC3220746

[B11] Hu K. Xie Y. Y. Zhang C. Ouyang D. S. Long H. Y. Sun D. N. (2012). MicroRNA expression profile of the hippocampus in a rat model of temporal lobe epilepsy and miR-34a-targeted neuroprotection against hippocampal neuron cell apoptosis post-status epilepticus. BMC Neurosci. 13:115 10.1186/1471-2202-13-115 22998082 PMC3471047

[B12] Hulse A. M. Cai J. J. (2013). Genetic variants contribute to gene expression variability in humans. Genetics 193, 95–108 10.1534/genetics.112.146779 23150607 PMC3527258

[B13] Iyer A. Zurolo E. Prabowo A. Fluiter K. Spliet W. G. van Rijen P. C. (2012). MiRNA-146a: a key regulator of astrocyte-mediated inflammatory response. PLoS ONE 7:e44789 10.1371/journal.pone.0044789 23028621 PMC3441440

[B14] Kostic A. D. Howitt M. R. Garrett W. S. (2013). Exploring host-microbiome interactions in animal models and humans. Genes Dev. 27, 701–718 10.1101/gad.212522.112 23592793 PMC3639412

[B15] Li J. Liu Y. Kim T. Min R. Zhang Z. (2010). Gene expression variability within and between human populations and implications toward disease susceptibility. PLoS Comput. Biol. 6:e1000910 10.1371/journal.pcbi.1000910 20865155 PMC2928754

[B16] Lukiw W. J. (2007). Micro-RNA speciation in fetal, adult and Alzheimer's disease hippocampus. Neuroreport 18, 297–300 10.1097/WNR.0b013e3280148e8b 17314675

[B17] Lukiw W. J. Pogue A. I. (2007). Induction of specific micro RNA (miRNA) species by ROS-generating metal sulfates in primary human brain cells. J. Inorg. Biochem. 101, 1265–1269 10.1016/j.jinorgbio.2007.06.004 17629564 PMC2080079

[B18] Lukiw W. J. Dua P. Pogue A. I. Eicken C. Hill J. M. (2011). Up-regulation of micro RNA-146a (miRNA-146a), a marker for inflammatory neurodegeneration, in sporadic Creutzfeldt-Jakob disease (sCJD) and Gerstmann- Straussler Scheinker (GSS) syndrome. J. Toxicol. Environ. Health 74, 1460–1468 10.1080/15287394.2011.618973 22043907 PMC3719866

[B19] Lukiw W. J. (2012a). NF-kB-regulated micro RNAs (miRNAs) in primary human brain cells. Exp. Neurol. 235, 484–490 10.1016/j.expneurol.2011.11.022 22138609 PMC3321120

[B20] Lukiw W. J. (2012b). Evolution and complexity of micro RNA in the human brain. Front. Genet. 3:166 10.3389/fgene.2012.00166 22969792 PMC3432495

[B21] Lukiw W. J. (2013). Antagonism of NF-κB-up-regulated micro RNAs (miRNAs) in sporadic Alzheimer's disease (AD); anti-NF-κB versus anti-miRNA strategies. Front. Genet. 4:77 10.3389/fgene.2013.00077 23641256 PMC3640190

[B22] McLachlan D. R. Lukiw W. J. Kruck T. P. (1989). New evidence for an active role of aluminum in Alzheimer's disease. Can. J. Neurol. Sci. 16, 490–497 2680008 10.1017/s0317167100029826

[B23] Nussbaum R. L. (2013). Genome-wide association studies, Alzheimer disease, and understudied populations. JAMA 309, 1527–1528 10.1001/jama.2013.3507 23571593

[B24] Olson M. V. (2012). Human genetic individuality. Annu. Rev. Genomics Hum. Genet. 13, 1–27 10.1146/annurev-genom-090711-163825 22657391

[B25] Pauling L. (1976). Vitamin C and the Common Cold. WH Freeman, San Francisco CA: WH Freeman Press.

[B26a] Raj A. Kuceyeski A. Weiner M. (2012). A network diffusion model of disease progression in dementia. Neuron 73, 1204–1215 10.1016/j.neuron.2011.12.040 22445347 PMC3623298

[B26] Raj A. Rifkin S. A. Andersen E. van Oudenaarden A. (2010). Variability in gene expression underlies incomplete penetrance. Nature 463, 913–918 10.1038/nature08781 20164922 PMC2836165

[B27] Reitz C. Jun G. Naj A. Rajbhandary R. Vardarajan B. N. Wang L. S. (2013). Variants in the ATP-binding cassette transporter (ABCA7), apolipoprotein E ϵ4, and the risk of late-onset Alzheimer disease in African Americans. JAMA 309, 1483–1492 10.1001/jama.2013.2973 23571587 PMC3667653

[B28] Saba R. Gushue S. Huzarewich R. L. Manguiat K. Medina S. Robertson C. (2012). MicroRNA 146a (miR-146a) is over-expressed during prion disease and modulates the innate immune response and the microglial activation state. PLoS ONE. 7:e30832 10.1371/journal.pone.0030832 22363497 PMC3281888

[B29] Sethi P. Lukiw W. J. (2009). Micro-RNA (miRNA) abundance and stability in human primary brain cells and in human brain tissues. Neurosci. Lett. 459, 100–104 10.1016/j.neulet.2009.04.052 19406203

[B30] Shadlen M. F. Larson E. B. Gibbons L. McCormick W. C. Teri L. (1999). Alzheimer's disease symptom severity in blacks and whites. J. Am. Geriatr. Soc. 47, 482–486 10203126 10.1111/j.1532-5415.1999.tb07244.x

[B31] Tang M. X. Stern Y. Marder K. Bell K. Gurland B. Lantigua R. (1998). The APOE-epsilon4 allele and the risk of Alzheimer disease among African Americans, whites, and Hispanics. JAMA 279, 751–755 10.1001/jama.279.10.751 9508150

[B32] Venketasubramanian N. Sahadevan S. Kua E. H. Chen C. P. Ng T. P. (2010). Interethnic differences in dementia epidemiology: global and Asia-Pacific perspectives. Dement. Geriatr. Cogn. Disord. 30, 492–498 10.1159/000321675 21252543

[B33] Wang X. Liu P. Zhu H. Xu Y. Ma C. Dai X. (2009). miR-34a, a microRNA up-regulated in a double transgenic mouse model of Alzheimer's disease, inhibits bcl2 translation. Brain Res. Bull. 80, 268–273 10.1016/j.brainresbull.2009.08.006 19683563

[B34] Williams R. B. Chan E. K. Cowley M. J. Little P. F. (2007). The influence of genetic variation on gene expression. Genome Res. 17, 1707–1716 10.1101/gr.6981507 18063559

[B35] Zhao Y. Bhattacharjee S. Jones B. M. Dua P. Alexandrov P. N. Hill J. M. (2013). Regulation of TREM2 expression by an NF-êB-sensitive miRNA-34a. Neuroreport 24, 318–323 10.1097/WNR.0b013e32835fb6b0 23462268 PMC4072209

[B36] Zheng W. Gianoulis T. A. Karczewski K. J. Zhao H. Snyder M. (2011). Regulatory variation within and between species. Annu. Rev. Genomics Hum. Genet. 12, 327–346 10.1146/annurev-genom-082908-150139 21721942

